# Phytobezoar: An Unusual Condition Leading to Small Bowel Obstruction

**DOI:** 10.7759/cureus.23885

**Published:** 2022-04-06

**Authors:** Ismail Aydin, Ilker Sengul, Demet Sengul

**Affiliations:** 1 General Surgery, Giresun University Faculty of Medicine, Giresun, TUR; 2 Endocrine Surgery-General Surgery, Giresun University Faculty of Medicine, Giresun, TUR; 3 Pathology, Giresun University Faculty of Medicine, Giresun, TUR

**Keywords:** pathology, surgical pathology, histopathology, emergency, bowel obstruction, intestines, small bowel, bowel, phytobezoar, bezoar

## Abstract

Bezoar is described as a swallowed, extraneous, and indigestible mass located in the gastrointestinal system; it accounts for 0.4-4.0% of all cases of mechanical intestinal obstruction. Intestinal obstruction is the most frequent complication of bezoar formation. Apart from intestinal obstructions, bezoars may also exhibit clinical symptoms such as abdominal pain, nausea, vomiting, weight loss, upper gastrointestinal bleeding, and gastric perforation. However, a considerable number of cases tend to be asymptomatic. Of note, its clinical symptoms cannot be differentiated easily from intestinal obstructions caused by other factors. As such, preoperative CT examination can provide invaluable information about the level of obstruction, etiology, and the existence of additional pathology and thereby help plan the type of surgical procedure required. If prompt diagnosis and timely treatment are not carried out, the condition may lead to significant morbidity and mortality.

## Introduction

Bezoars account for approximately 0.4-4.0% of all cases with mechanical intestinal obstruction. The word “bezoar” originates from the Arabic word “bedzehr” or the Persian word “padzhar,” which means “protecting against a poison.” Bezoars, defined as a swallowed, extraneous, and indigestible mass in the gastrointestinal system, are classified into different types according to undigested accumulative agents that they originate from: (i) pharmacobezoars, developing due to certain medications, (ii) phytobezoars, caused by the indigestible components of fibrous foods and fruits, (iii) lactobezoars, originating from lactoproteins, and (iv) trichobezoars, developing due to swallowed hair, pilar, and cilium. Some bezoars occur due to the swallowing of plastic and metal pieces. In addition, Rapunzel syndrome has been determined to be an unusual form of bezoar extending from the stomach to the small intestine or beyond [1-3]. Phytobezoar can be caused by the consumption of an excessive amount of cabbage, wheat, corn, grape skin, potato, seeds of the fruit, fig, and green-leaved vegetables. It may present with a stomachache, bleeding, anorexia, vomiting, weight loss, dysphagia, palpable mass, diarrhea or constipation, intestinal obstruction, and even intestinal perforation, or just might remain asymptomatic for a long period. Phytobezoar-induced small bowel obstruction is an uncommon entity and is usually removed via a surgical approach [4,5]. Imaging findings are invaluable for the diagnosis of bezoar, and sonographic findings are routinely used for the diagnosis even though they are associated with some disadvantages. CT is superior to other imaging tools in the diagnosis of bezoar and the relevant differential diagnosis with regard to intestinal obstruction. Besides determining the localization and the level of obstruction, CT has also been utilized to identify intestinal ischemia and potential intestinal diseases [6,7]. Early and urgent action might be crucial to avoid complications and mortality associated with the condition. In this report, We discuss a case of phytobezoar presenting with symptoms of acute mechanical intestinal obstruction and describe the approach to its diagnosis and treatment.

## Case presentation

**Figure 1:**
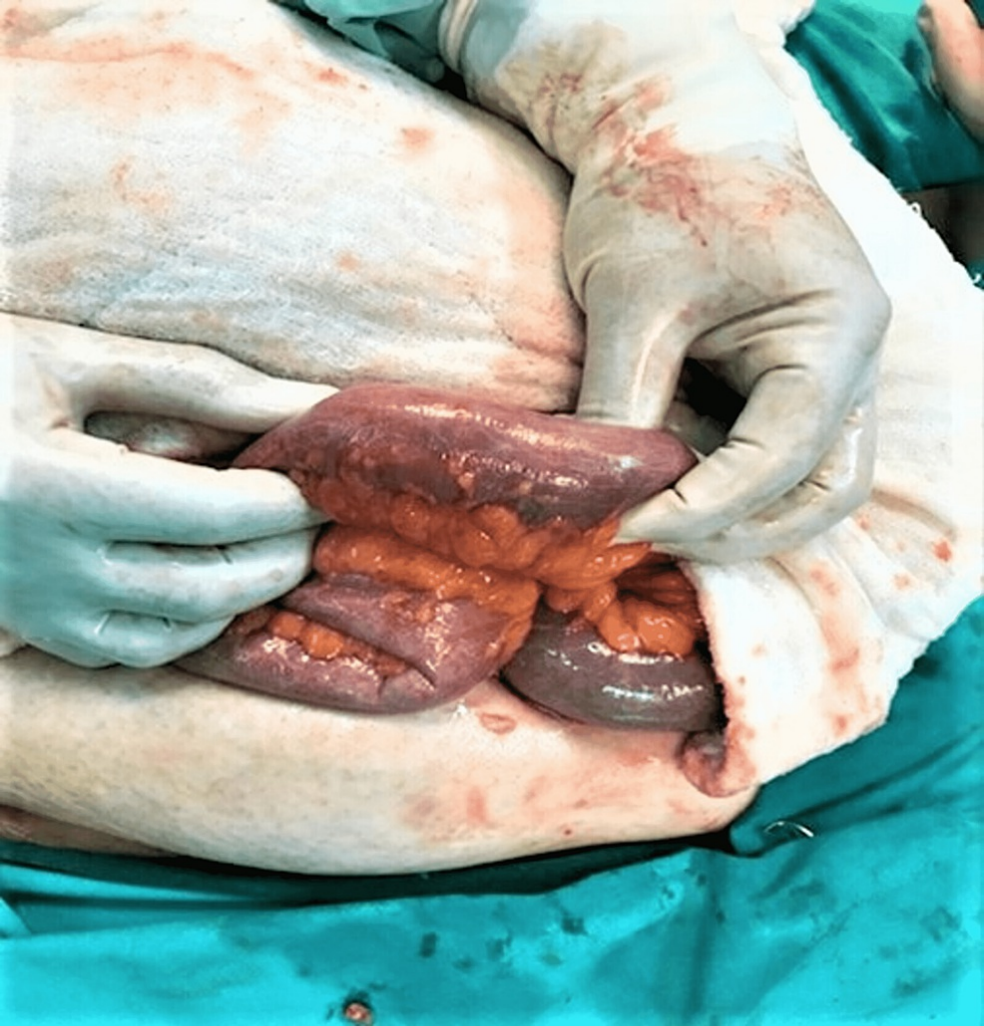
The phytobezoar is seen completely obstructing the small bowel lumen

**Figure 2:**
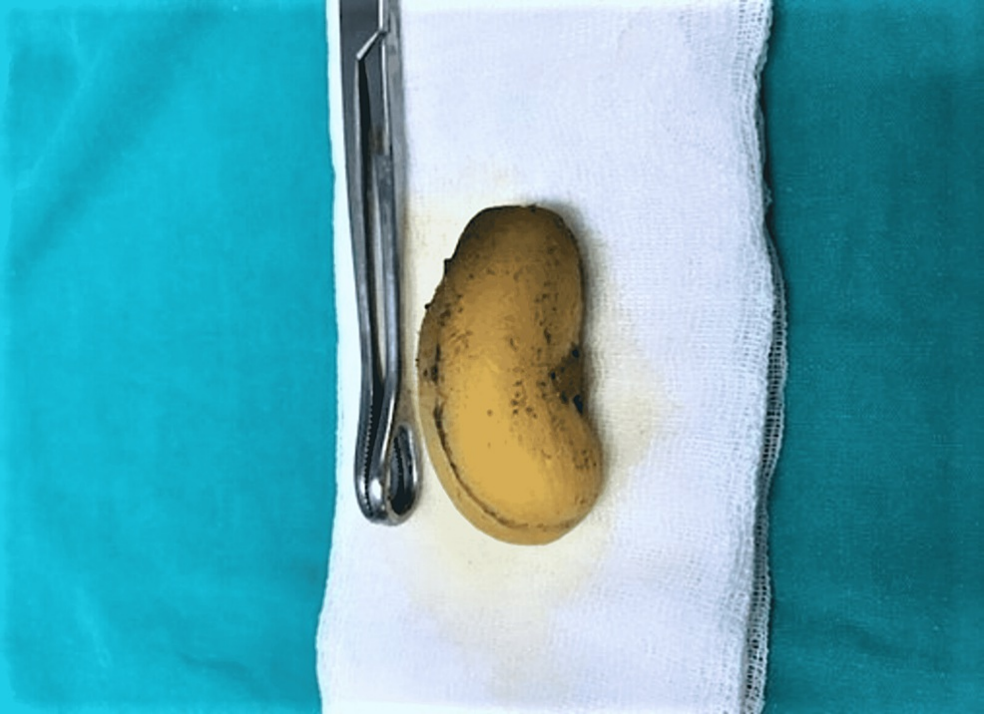
The phytobezoar, an entirely swallowed apricot fruit, is removed from inside of the small bowel

The patient was an 82-year-old female who presented to the emergency service with complaints of stomachache, nausea, abdominal swelling, and inability to defecate for the last three days. On review, her personal medical history was remarkable for cholecystectomy and acromphalus. On physical examination, tenderness in the abdomen and abdominal distension were recognized. Imaging studies revealed intestinal air-liquid levels on the abdominal X-ray and a mass-like formation that filled the lumen in the distal ileal segment leading to the dilatation in the proximal loops (raising suspicion for intussusception or bezoar) on contrast-enhanced abdominal CT. She underwent emergency surgery and the laparotomy exhibited a mass compatible with bezoar that completely obstructed the intestinal lumen, approximately 50 cm proximal from the ileocecal valve (Figures 1, 2). The emerged bezoar was removed by performing enterotomy, following the primary bowel repair. The patient recovered uneventfully and was discharged home on hospital day five.

## Discussion

It has been reported that the rates of obstruction due to the bezoars are between 0.4 and 4%. Bezoars tend to develop in both the stomach and the small intestine. Moreover, intestinal obstruction is the most common type of complication of bezoar formation [7,8]. Additionally, phytobezoar, the most common type of bezoar, may lead to obstruction of the small intestine at a rate of approximately 4% [4,9]. Bezoar formation is not directly related to gender and age and can be affected by various factors such as diet, anatomic features, an organic compound of the gastrointestinal system, and functional disorders [10,11]. In terms of diet, phytobezoar results from excessively consuming products such as persimmon, hawthorn, date palm, dry prune, grapes, celery, mangoes, bananas, cactus fruits, figs, and mushrooms, which contain indigestible plant fibers [12].

Even though regional and seasonal variations can be observed, bezoars might affect people all around the world. Obstruction is generally seen in the narrow part of the small intestine, particularly 50-70 cm distant from the ileocecal valve since the intestinal lumen is narrow at that level. The second most common region is jejunum [13]. The patient in the present study reported that she had swallowed a whole apricot fruit. We postulate that the obstruction might have occurred due to that. The localization of obstruction was also approximately 50 cm proximal from the ileocecal valve, consistent with the literature. Although bezoars, apart from intestinal obstruction, can exhibit clinical symptoms such as abdominal pain, nausea, vomiting, weight loss, gastrointestinal system bleeding, and stomach perforation, a considerable number of cases may remain asymptomatic. Iwamuro et al. [1] have reported on five cases (16,2%) that were asymptomatic and bezoars were determined by chance in those in their series of 31 cases.

Abdominal roentgen, barium enema, ultrasound, and endoscopy can be performed in order to establish a diagnosis of obstruction due to a bezoar. However, those methods may be inadequate and have several limitations [10]. In intestinal obstruction, in addition to the bezoar, a CT scan is the most effective method to determine the accurate treatment modality for the detection of co-existing multiple bezoars, intestinal ischemia, perforation, and other potential intestinal diseases [6,10]. The most frequently seen CT findings of bezoars include the appearance of a round or ovoid or a long sausage-shaped mass containing mottled gas at the obstructed site. In rare cases, phytobezoar may resemble a gasless soft tissue mass, an intraluminal tumor, or intussusceptions [13]. Endoscopy can assist with both the diagnosis and the treatment of bezoars with proximal localization [14]. Several studies have reported that bezoars with proximal localization may be removed endoscopically and that method should be tried as the primary intervention [15]. To this end, definitive surgical treatment is required for bezoars that obstruct the small intestine. Conventional or laparoscopic surgical approaches can be planned in order to resolve these issues. The most frequently employed procedure is to remove the bezoar with enterotomy. In our case, we opted for removing the bezoar by enterotomy, following the primary bowel repair. Last but not least, histopathologically, delayed surgery may be associated with more inflammatory alterations. In addition, these inflammatory alterations might lead to some irreversible cytostructural and histopathologic changes in the relevant tissues, cyto- and/or histopathologically [16]. Therefore, emergency surgery remains significant and critical, globally, in severe cases [17-20].

## Conclusions

Phytobezoar-induced intestinal obstruction remains an uncommon etiology of small bowel obstruction. This phenomenon, per se, may appear in the absence of previous gastrointestinal surgery and might easily be overlooked without a high index of clinical suspicion. Physicians should focus on a detailed dietary history, contributing factors, and imaging features. The rates of mobility and mortality may be attenuated in these cases through a rapid and effective approach, which involves immediate decision-making and opting for an appropriate surgical procedure. Bene diagnoscitur, bene curatur.
